# Stacked neural network for predicting polygenic risk score

**DOI:** 10.1038/s41598-024-62513-1

**Published:** 2024-05-21

**Authors:** Sun bin Kim, Joon Ho Kang, MyeongJae Cheon, Dong Jun Kim, Byung-Chul Lee

**Affiliations:** Genoplan Korea Inc., Seoul, Republic of Korea

**Keywords:** Polygenic risk score, Deep learning, Ensemble learning, Computational models, Bioinformatics

## Abstract

In recent years, the utility of polygenic risk scores (PRS) in forecasting disease susceptibility from genome-wide association studies (GWAS) results has been widely recognised. Yet, these models face limitations due to overfitting and the potential overestimation of effect sizes in correlated variants. To surmount these obstacles, we devised the Stacked Neural Network Polygenic Risk Score (SNPRS). This novel approach synthesises outputs from multiple neural network models, each calibrated using genetic variants chosen based on diverse p-value thresholds. By doing so, SNPRS captures a broader array of genetic variants, enabling a more nuanced interpretation of the combined effects of these variants. We assessed the efficacy of SNPRS using the UK Biobank data, focusing on the genetic risks associated with breast and prostate cancers, as well as quantitative traits like height and BMI. We also extended our analysis to the Korea Genome and Epidemiology Study (KoGES) dataset. Impressively, our results indicate that SNPRS surpasses traditional PRS models and an isolated deep neural network in terms of accuracy, highlighting its promise in refining the efficacy and relevance of PRS in genetic studies.

## Introduction

Significant scientific and biological findings have been fueled by genome-wide association studies (GWAS), with these studies highlighting a myriad of associations between intricate traits and common genetic variants within populations^1^. As larger sample sizes have been used in more recent GWAS, the associations between various diseases or traits have been revealed more accurately and distinctively^1^. A probabilistic measure known as the polygenic risk score (PRS) can predict an individual’s disease susceptibility from GWAS outcomes. Additionally, PRS contributes to our understanding of genetic infrastructure and assists in making clinical decisions, specifically in areas such as risk stratification, early disease detection, and the prevention of prevalent adult-onset diseases^2–5^.

Unadjusted PRS were initially derived from statistically significant GWAS variants in the early stages of PRS development^6^. Overfitting was a notable risk when dealing with small sample sizes or a high variant count^7–9^. To address the challenges of genetic variant analysis, statistical regularisation models like BLUP were employed^10,11^. However, these models might overestimate the effect size of correlated variants. Emerging models like BayesR^12^, framed as an individual-level Hierarchical Bayesian Mixture Model, offer a refined approach to genetic variant analysis.

In the effort to manage genetic overestimation, the concept of linkage disequilibrium (LD) has been crucial. By adopting a strategy of selecting representative single nucleotide polymorphisms (SNPs) within LD blocks, or P+T, significant genetic variants are identified based on a specific p-value threshold. However, such a selection process is potentially biased. The P+T method, which selects the most representative variants within a predetermined size and strand shifting window, may introduce bias in variant selection and fails to accurately reflect the real effect size of variants that are correlated with other variants. To mitigate this, LD panels have been utilised, allowing for the prediction of the effect size of all variants on the traits. The development of methods like LDpred^13^, Lassosum^14^, PRS-CS^15^, and SBayesR^16^ emphasizes the evolving strategies in this field.

While these methods offer notable advantages, they are not without limitations. One key issue is the necessity for costly LD reference panels, which often lack ancestral diversity. Moreover, these models might struggle to accurately depict real-world scenarios, particularly when non-linear effects and interactions come into play. LD panels are assembled block-wise, implying that correlations are only considered among variants within the same block. In the continued evolution of PRS methodologies, NeuPred^17^ stands out. Utilising neuronised priors, the model further refines predictions by employing a cross-validation strategy for prior selection. This rigid structure may unintentionally bypass valid genetic variants that exhibit non-linear effects or interact with other variants, leading to an incomplete picture of genetic influences.

The ensemble machine learning method of stacking^18^, presents a robust solution for harnessing a broader range of variants. This methodology involves creating a final decision model by using the outputs of multiple models as inputs. Within this context, Prive et al.^19^ proposed a novel technique, Stacked Clumping and Thresholding (SCT). The SCT is a unique polygenic score derived from stacking all P+T scores and adjusting various hyperparameters including p-value, window size, and correlation thresholds. Unlike traditional methods that select one set of hyperparameters to maximize prediction in a training set, SCT takes a different approach. It employs an efficient penalised regression to learn an optimal linear combination of all P+T scores. This innovative technique extends the ability to optimize prediction beyond the limitations of a single training set.

Conventional PRS models, derived from GWAS effect sizes, largely operate under an additive model assumption^20^. This approach may overlook the nuances of other genetic architectures such as the multiplicative model^21^. Furthermore, the lack of consideration for interactions between genetic variants, such as epistasis^22,23^, constrains these models. Such limitations potentially bypass the detection of complex genetic phenomena, failing to account for variants that exert non-linear or interactive influences on the manifestation of traits or diseases. Consequently, the capabilities of conventional PRS models to accurately predict phenotypic outcomes may be hampered.

Predicting polygenic risk with machine learning models offers a way to encapsulate variant interactions and non-linear effects, with fewer presuppositions about the genetic architecture^24^. A deep neural network (DNN)-based PRS model for breast cancer demonstrated superior performance compared to other machine learning models and conventional PRS models^24^. Similarly, a study that utilised DNN models for predicting Alzheimer’s disease PRS surpassed the efficacy of conventional PRS models and graph neural network (GNN) models^25^. Despite the commendable performance of DNN, it was necessary to determine a specific p-value to select valid genetic variants before building the model because of the curse of dimensionality^8^.

In an endeavour to more accurately capture the non-linear effects and broad-spectrum of genetic variants, we propose a novel approach: the Stacked Neural Network Polygenic Risk Score (SNPRS). The SNPRS model utilises the principles of ensemble learning^26^, combining outputs from multiple neural network models. These models are trained on genetic variants chosen across a broad spectrum of p-value thresholds, representing a departure from conventional practices that rely on predetermined p-value thresholds to select significant genetic variants. This strategy reduces the risk of overlooking key variants with non-linear effects, allowing us to more accurately determine the effect size of variant combinations that might otherwise be lost in hard thresholding. We put our newly proposed SNPRS to the test using real data sourced from the UK Biobank (UKBB). Our objective was to predict the genetic risk of gender-specific cancers, specifically breast cancer and prostate cancer. To evaluate the performance of SNPRS, we employed metrics such as Nagelkerke R-square and the Area Under the Receiver Operating Characteristic curve (AUC ROC). Expanding our scope, we also applied SNPRS to quantitative traits such as height and BMI, using r-square for evaluation. Furthermore, to test the robustness of our model across different populations, we utilised data from the Korea Genome and Epidemiology Study (KoGES)^27^, representing an East Asian cohort. Our findings demonstrate that SNPRS outperforms traditional models such as P+T, PRSice, and the BayesR model, as well as more recent models like PRS-CS that utilize LD reference panels. Furthermore, SNPRS was shown to be superior to a single DNN model trained with variants filtered based on a specified p-value. In this light, SNPRS emerges as a robust and reliable model for predicting genetic risk, enhancing the predictive accuracy and relevance of polygenic risk scores in genetic research.

## Results

### Overview of methods

**Figure 1:**
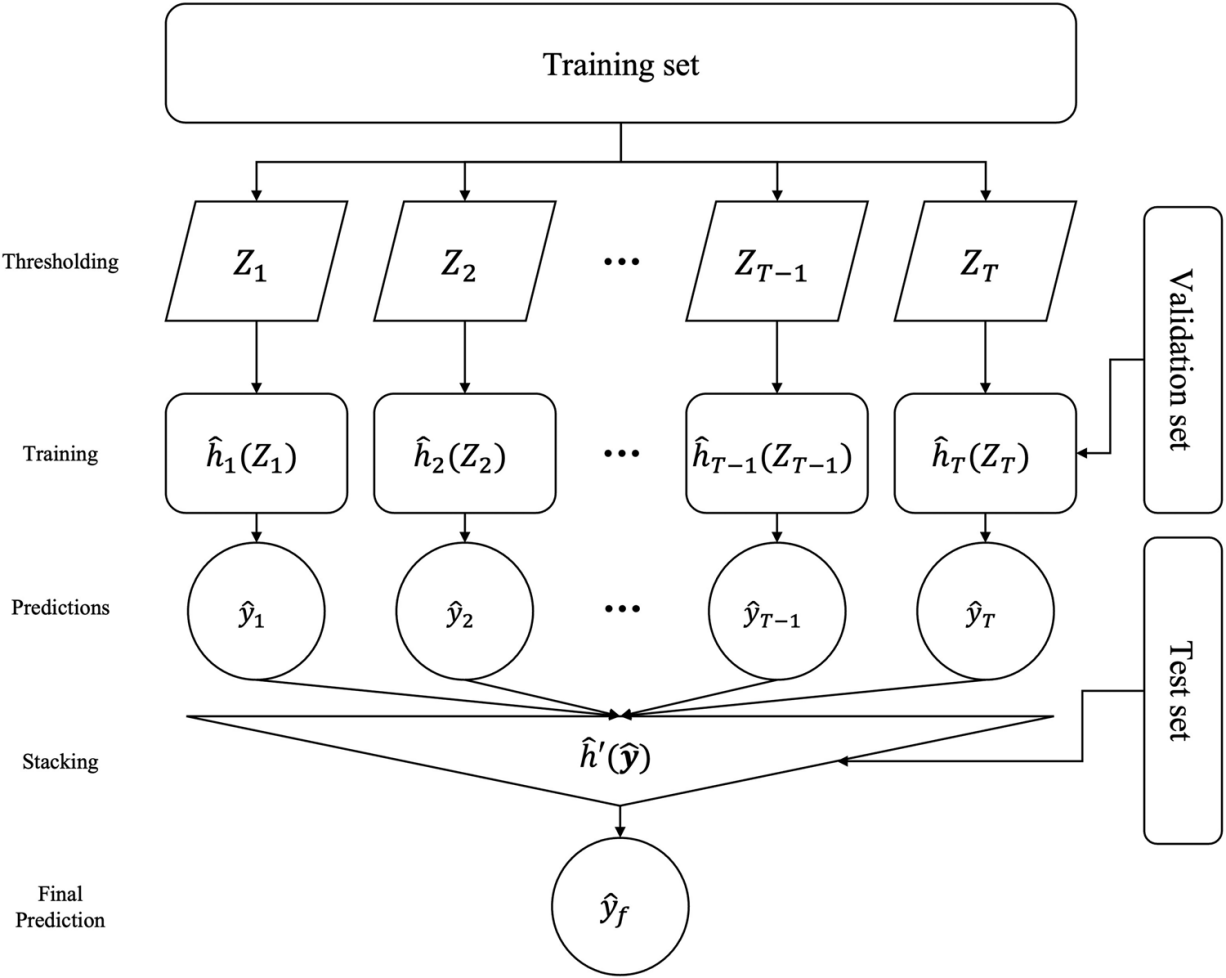
The overall architecture of SNPRS model.

We utilised the stacking method to regularize our DNN models, an approach that helps to alleviate the overfitting often associated with stringent thresholding. We embarked on the training of multiple DNN models, each operating with a unique set of genetic variants filtered according to varying p-value thresholds $p_{t}$. The stacked model $\hat{H}$ was constructed as per Fig. 1 and Eq. (1), wherein $\hat{h'}$ serves as an aggregator, $\hat{h}_{t}$ denotes the base DNN models, and $Z_{t}$ represents a genotype-individual matrix filtered in line with the p-value $p_{t}$.

1 $$\begin{aligned} {\hat{H}(Z_{1:T}) = \hat{h}'(\hat{h}_{1}(Z_{1}),\hat{h}_{2}(Z_{2}),\dots ,\hat{h}_{T}(Z_{T}))} \end{aligned}$$

The hyperparameters $\hat{h}_{t}$ for the DNN models were fine-tuned using a grid search methodology in conjunction with a validation set. Subsequently, the outputs from the top-performing models for each dataset—filtered according to respective p-value thresholds—were aggregated. This cumulative output served as the foundation for training our stacked neural network model. This model was designed to discern the optimal weighting for the output of each individual model, thereby enhancing the accuracy and efficacy of the final decision-making process.

### GWAS

**Table 1:** Qualitative traits information.

Dataset	Trait	Cases	Controls	Imputed SNPs	Imputed $\cap$ 1KG SNPs	Array SNPs
UKBB	Breast cancer	9285	95,140	6,575,143	749,769	477,359
UKBB	Prostate cancer	7727	87,014	6,575,143	749,769	477,359
KoGES	Breast cancer	399	36,725	7,949,945	992,787	–
KoGES	Prostate cancer	39	19,713	7,949,945	992,787	–

**Table 2:** Quantitative traits information.

Dataset	Trait	Samples	Imputed SNPs	Imputed $\cap$ 1KG SNPs	Array SNPs
UKBB	Height	499,876	6,575,143	749,769	477,359
UKBB	BMI	499,310	6,575,143	749,769	477,359
KoGES	Height	58,692	7,949,945	992,787	–
KoGES	BMI	58,688	7,949,945	992,787	–

We undertook a GWAS on the UKBB which we partitioned into training, validation, and test sets. For UKBB, the GWAS was performed on the training set, incorporating both array data and the imputed dataset. Our analyses were substantiated by Manhattan and Q–Q plots, which visually demonstrated the significance and distribution of variants relative to the trait under examination. The KoGES dataset was not utilised for GWAS; instead, it served solely as an external dataset for testing the performance of the models.

The array dataset was primarily used for our proposed SNPRS and DNN models. On the other hand, the imputed datasets, which were generated by inferring genotypes at unobserved loci using known LD information, were employed for the conventional models. This approach enhanced the granularity and comprehensiveness of the dataset used for the conventional models. Further details about the sample size and SNP count are provided in Tables 1 and  2. The figures in the “Imputed $\cap$ 1KG SNPs” columns in Tables 1 and  2 represent the number of SNPs, specifically those that intersect between the imputed SNPs and the 1KG reference panel used for the PRS-CS model. These tables illustrate the overall sample sizes, not exclusively those from GWAS.

Manhattan plots and Q–Q plots as shown in Supplementary Figs. 1–4, displayed the statistical significance of genetic variants throughout the genome, underscoring the successful identification of relevant associations in our GWAS within the training set. The imputed dataset exhibited a larger number of significant variants within identical LD blocks, suggesting their potential genetic relevance.

### Polygenic risk score

**Figure 2:**
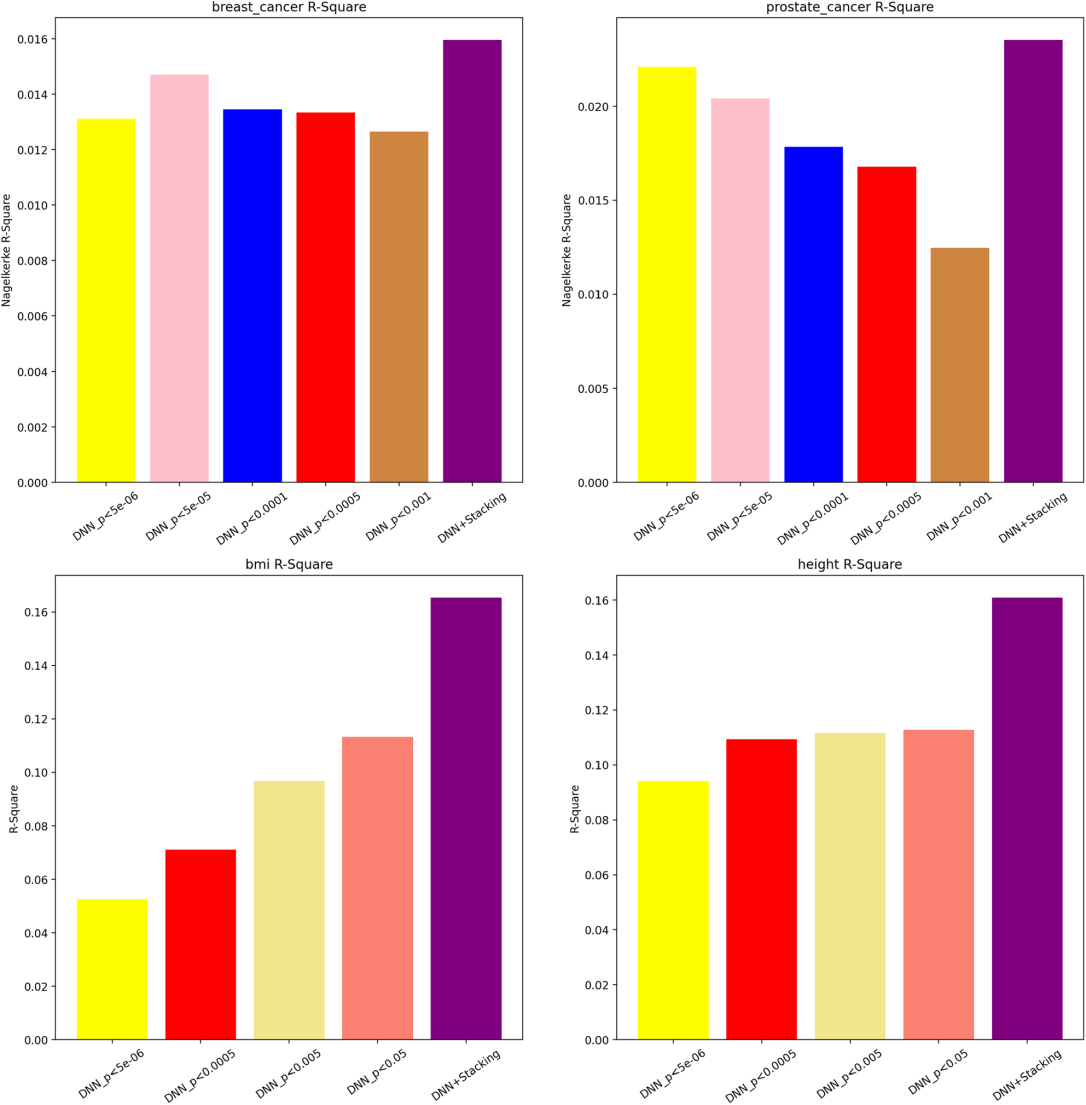
Nagelkerke R-square and R-square plot comparison for UKBB dataset with single models and stacked one.

**Figure 3:**
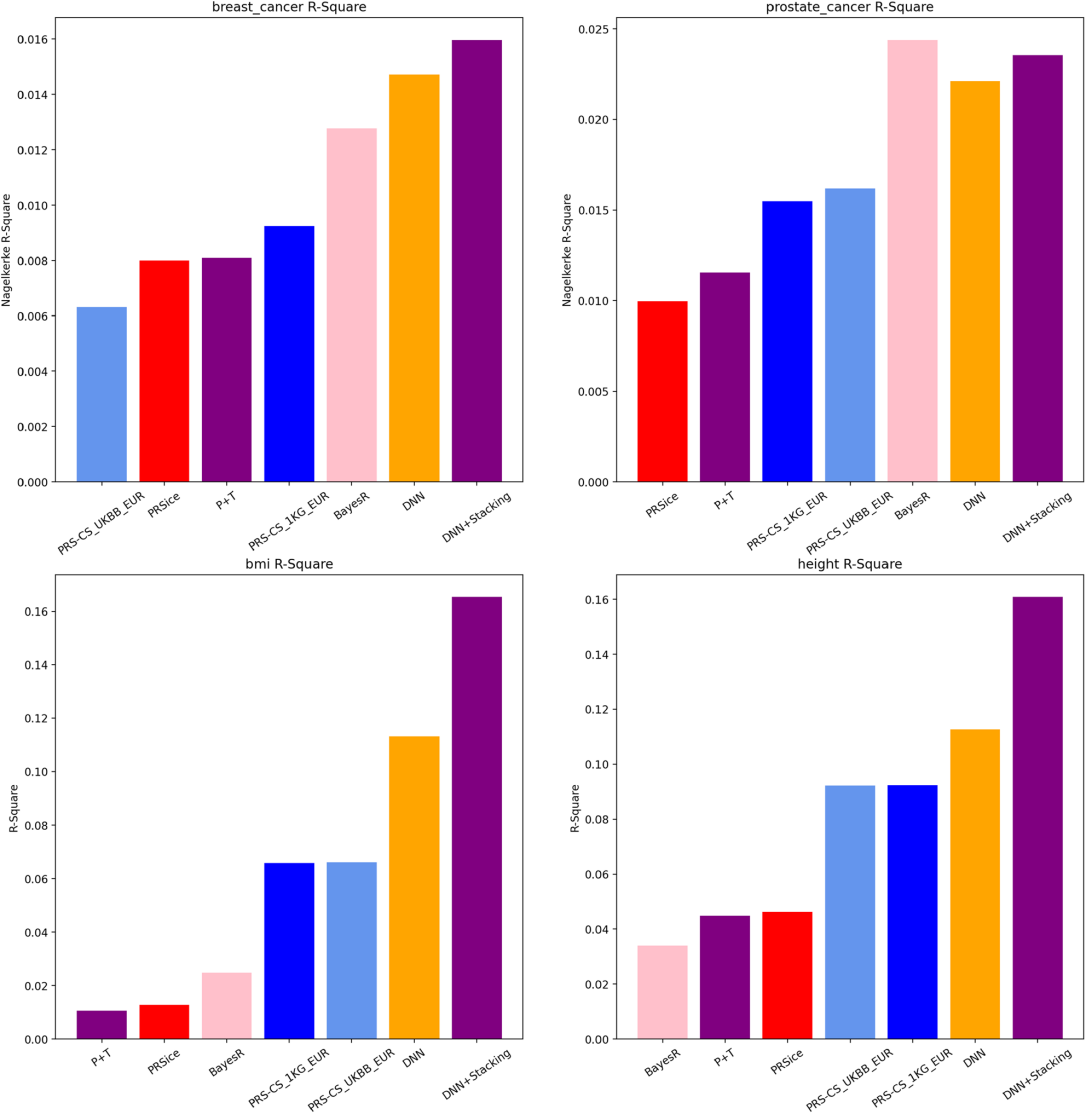
Nagelkerke R-square and R-square plot comparison for UKBB dataset with other models.

**Figure 4:**
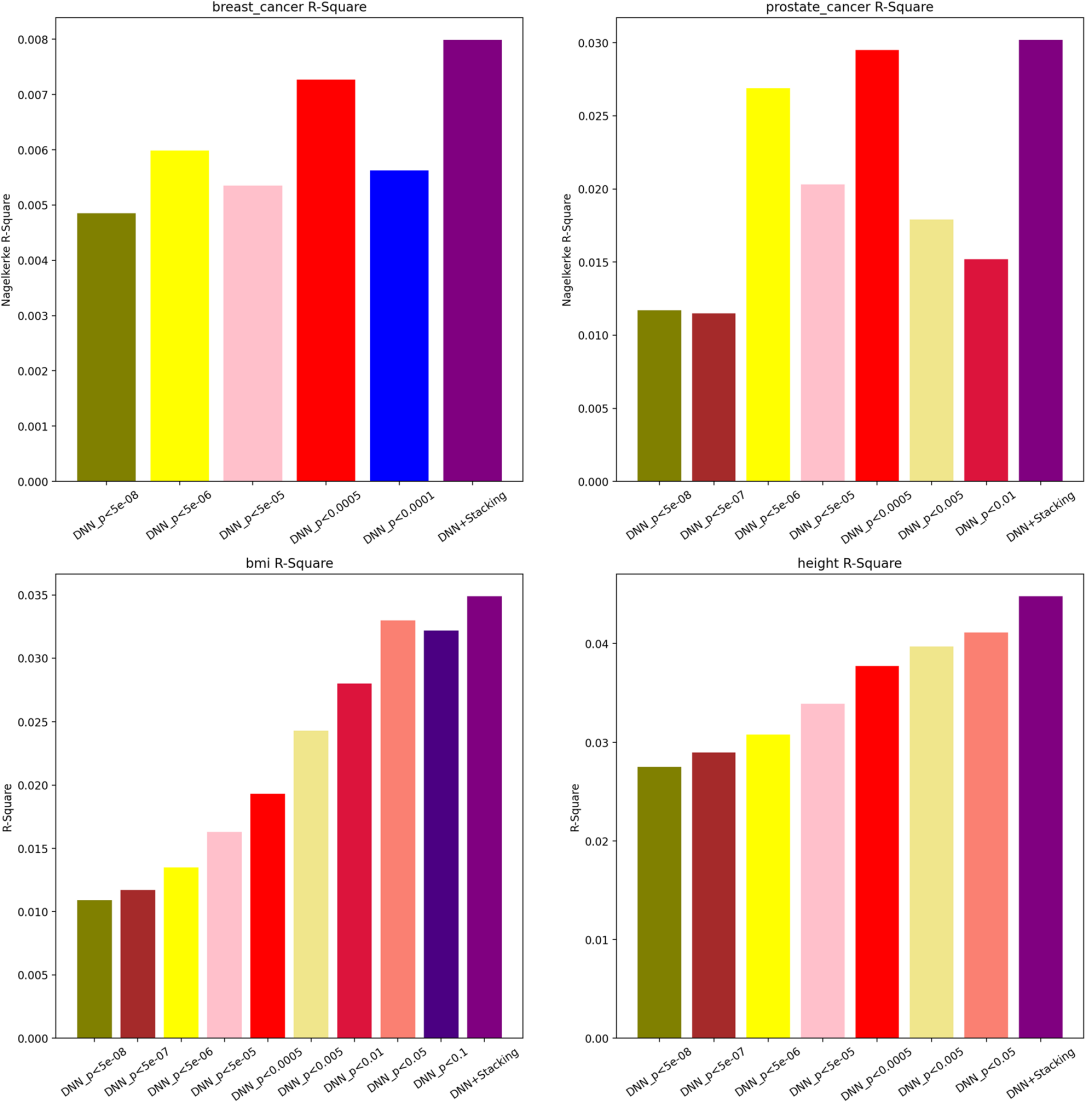
Nagelkerke R-square and R-square plot comparison for KoGES dataset with single models and stacked one.

**Figure 5:**
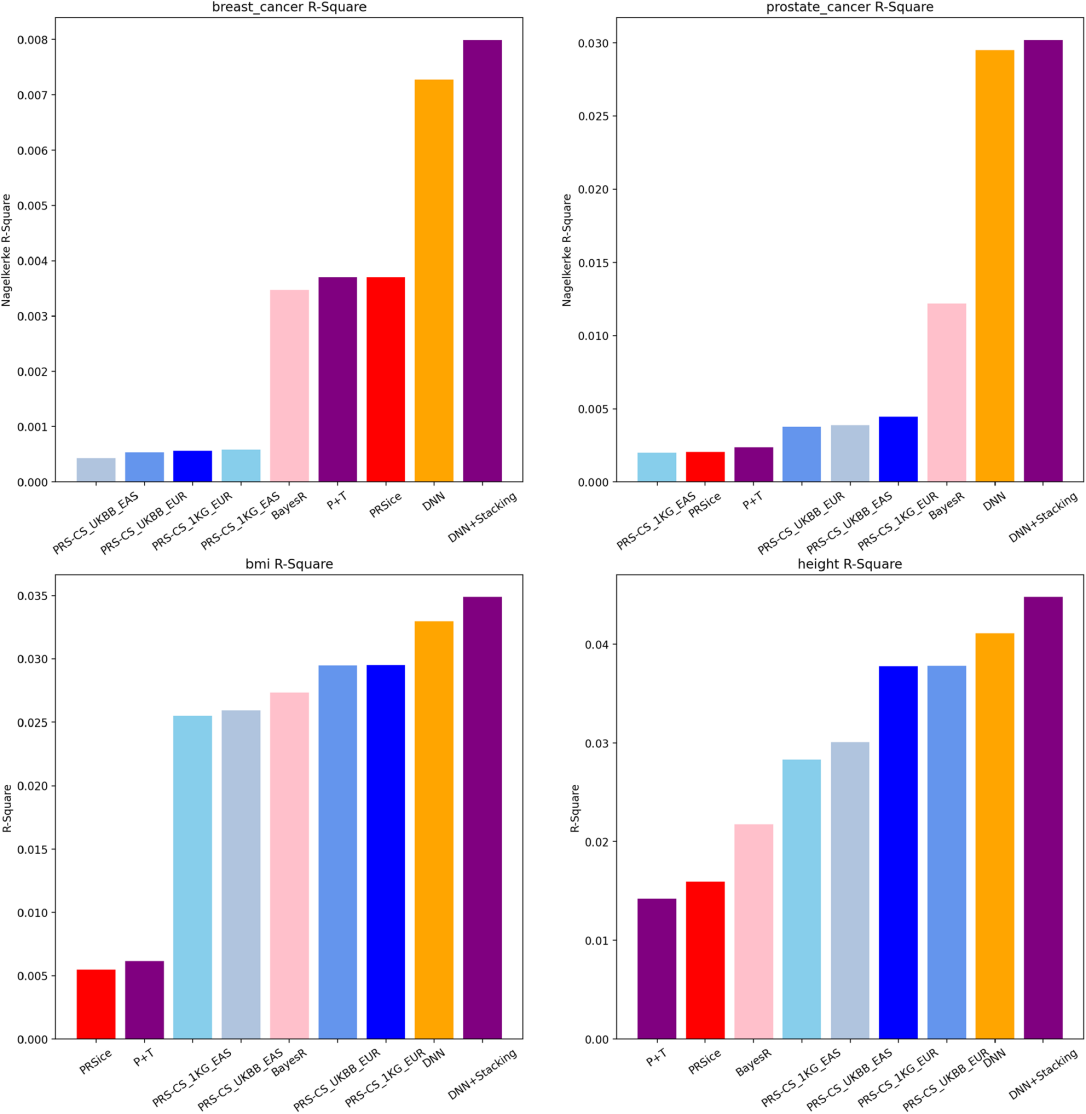
Nagelkerke R-square and R-square plot comparison for KoGES dataset with other models.

#### Qualitative traits

Our objective was to investigate the capabilities of the SNPRS model in accurately predicting gender-specific cancers such as breast and prostate cancer. We sought to achieve this by benchmarking the performance of SNPRS against conventional models as well as DNN models that employ a single statistical standard. Traditional baseline models included P+T, PRSice, BayesR and PRS-CS, each with two LD panels, 1KG and UKBB. Performance evaluations were carried out through Nagelkerke R-square and ROC curve AUC metrics. The graphical representations of these findings can be seen in Fig. 3 and Supplementary Figs. 5 and 6. Conventionally, we implemented the imputed UKBB genotype data, which is commonly used in model building utilising imputed genotype data summary statistics. Conversely, in our application of DNN models, we used UKBB array genotype data during GWAS to decrease feature dimensions. This strategy was effective in avoiding features having highly correlated variants that showed statistical significance to the trait under consideration. Subsequently, we utilised GWAS results for selecting significant variants with a set range of p-value thresholds in the DNN model.

Our initial point of comparison was the performance of DNN models applying hard thresholding versus the SNPRS model. The Nagelkerke R-square results, depicted in Fig. 2, reveal that the SNPRS model outperformed the DNN models with hard thresholding across the board. In the case of breast cancer, a trend was observed where the model performance improved as the p-value threshold decreased. However, a steep drop in performance was observed at the p-value threshold of 5e−06, with the Nagelkerke R-square reaching approximately 0.013. This value was lower than the peak performance achieved at the p-value threshold of 5e−05, which was marginally less than 0.015. For prostate cancer, the trend varied. As the p-value threshold decreased, a steady and more significant increase was observed in the model performance, without any observable decline, unlike in the breast cancer model. Regardless of the trait considered, the SNPRS model consistently outshined the DNN models that were trained with a single set of variants. Specifically, SNPRS models obtained 8.5% and 6.4% higher scores for breast cancer and prostate cancer, respectively. AUC results, displayed in Supplementary Figs. 5 and 6, mirrored the trend observed in the Nagelkerke R-square, albeit on a smaller scale. For breast cancer, the DNN model trained with a p-value threshold of 5e−05 attained the highest AUC. However, the AUC of the DNN model trained with a more stringent p-value threshold of 5e−06 was slightly lower, paralleling the trend in Nagelkerke R-square. In the context of prostate cancer, better performance was associated with stricter p-value thresholds. Similar to the Nagelkerke R-square results, the SNPRS model also surpassed all other models trained with a single p-value threshold in terms of AUC, further cementing its superior performance in predicting gender-specific cancers.

When surveying the conventional models, the nuances in their performances were hard to overlook. For breast cancer, PRSice’s Nagelkerke R-square score stood at 7.998e−3, just behind the P+T model, which achieved a score of 8.107e−3. The difference, although slender at about 1.4%, highlights the intricate disparities between the models. The PRS-CS models, when segregated by their LD reference panels, further underscored this point. Specifically, the PRS-CS model with the 1KG panel eclipsed its UKBB counterpart in breast cancer predictions by an impressive margin of 29.5%. Transitioning to prostate cancer, PRSice achieved a score of 9.972e−3, trailing the P+T model by roughly 15.9%. Interestingly, the PRS-CS model’s dynamics shifted, with the UKBB panel variant outshining the 1KG variant by about 4.5%. However, the BayesR model emerged as the crown jewel among conventional models, especially in prostate cancer, registering a commendable Nagelkerke R-square score of 2.438e−2. On the AUC front, the differences further elucidated the models’ relative competencies. In breast cancer, BayesR achieved a score of 0.5735, bettering the PRS-CS with 1KG panel model, which managed a score of 0.5636, by 1.75%. For prostate cancer, BayesR’s robust performance continued, scoring 0.6047, which was approximately 3.3% superior to the next best conventional model, PRS-CS with UKBB panel. Shifting our gaze to the more advanced DNN model, it distinctly asserted its superiority over conventional counterparts. When assessing breast cancer predictions, the DNN model’s Nagelkerke R-square score surpassed the best conventional model, PRS-CS with 1KG panel, by an astounding 59.6%. The AUC narrative was consistent; the DNN model’s score was a testament to its predictive prowess over the conventional algorithms. Prostate cancer predictions, though, saw the BayesR model challenging this dominance. Despite BayesR’s impressive numbers, the DNN model’s Nagelkerke R-square score remained ahead of all conventional models except BayesR, exhibiting a notable 27.8% lead over the PRS-CS with UKBB panel model. Our primary focus, the DNN+Stacking model, showcased its capabilities emphatically. In breast cancer predictions, this model trumped the single DNN model in Nagelkerke R-square scores by about 8.6%. In terms of AUC, the superiority of the DNN+Stacking model remained evident. However, prostate cancer brought forth a more riveting contest. The DNN+Stacking model’s R-square score of 2.3536e−2 was approximately 2.5% ahead of the single DNN model. But in a tight race, the BayesR model slightly outperformed the DNN+Stacking model by around 3.5%, although the latter still made a significant leap over other conventional models. In AUC terms, for prostate cancer, the BayesR model’s performance of 0.6047 was distinctively ahead, signaling its efficacy in this particular prediction scenario. Still, the DNN+Stacking model proved to be an innovative approach, showing immense promise, especially when contrasted with traditional models.

#### Quantitative traits

In our comprehensive exploration of the predictive capacities of various models for two quantitative traits, height and BMI, for the UKBB dataset, discernible patterns emerged. These patterns, as showcased in Figs. 2 and  3, provide critical insights into the underlying dynamics. The subtleties of SNP selection, influenced by shifting p-value thresholds, significantly impact the efficacy of deep neural network models. For instance, within the UKBB dataset related to height, as the threshold became more stringent from 0.05 to 5e−06, the R^2^ values observed a decrease of approximately 16%. However, when the deep neural network model was enhanced with stacking techniques, there was an approximately 42% increase in R^2^ from the basic DNN model’s value at the most liberal threshold, underscoring the advantages of using hybrid methodologies. A similar trend is visible for BMI within the UKBB cohort. The R^2^, starting at 0.1133 with a threshold of 0.05, witnessed a decline of nearly 54% as the threshold tightened to 5e−06. Nevertheless, the combined DNN model with stacking showcased an R^2^ value that was approximately 46% higher than the base DNN model’s initial value.

When juxtaposing different models within the UKBB dataset, particularly for height, the DNN+Stacking model displayed an R^2^ value that was about 43% higher than the standalone DNN model. Traditional models like P+T, PRSice, and BayesR yielded values that were roughly 64% lower than the DNN+Stacking model. A similar distribution was observed for BMI metrics within the UKBB, where the DNN+Stacking’s R^2^ value was about 46% higher than the standalone DNN model.

#### Validation with external dataset

Our analysis also included results from the KoGES. Adding the KoGES data to our evaluation provided a broader context for our findings, allowing us to assess the models’ performance across a more diverse dataset. This inclusion emphasised the robustness of the SNPRS model in various genetic backgrounds and further validated the superior performance of the DNN+Stacking model, where it outperformed the single DNN model in both Nagelkerke R-square and AUC scores.

For breast cancer, the ROC AUC results in Supplementary Figs. 7 and 8 show that the DNN+Stacking model achieves the highest AUC (0.5926), indicating a strong predictive performance. This model outperforms other DNN models across various p-value thresholds, with the closest being the DNN model at a p-value threshold of <0.0005 (AUC = 0.5891). Among the conventional models, BayesR displays a competitive AUC (0.5588), closely followed by the P+T model (AUC = 0.5688). The PRS-CS models with different ethnic LD reference panels show relatively lower performances, with the European panel (PRS-CS_1KG_EUR) achieving an AUC of 0.5249 and the East Asian panel (PRS-CS_1KG_EAS) slightly higher at 0.5266. In prostate cancer predictions, the DNN+Stacking model again shows its superiority with the highest AUC (0.6631) as visualised in Supplementary Figs. 7 and 8. The DNN models with varying p-value thresholds demonstrate a trend where stricter thresholds correlate with improved AUC scores, with the model at p-value <0.01 achieving an AUC of 0.6408. The traditional models perform less well in comparison, with BayesR leading (AUC = 0.6131) followed by the P+T model (AUC = 0.5669).

The Nagelkerke R-square results in Fig. 5 for breast cancer show that the DNN+Stacking model achieves the highest R-square value (7.99e−3), indicating a robust variance explanation capability. This model’s performance is closely followed by the DNN model at p-value <0.0001. The conventional models, while trailing behind the DNN models, exhibit a modest variance explanation, with BayesR and the P+T models showing the highest R-square values among them. In the case of prostate cancer, as shown in Fig. 4, the DNN+Stacking model also achieves the highest R-square value (3.02e−2), which is considerably higher than that of the single DNN models across various p-value thresholds. The conventional models show lower performance, with the BayesR model achieving the highest R-square value among them (1.21e−2), followed by the PRS-CS with the 1KG panel (4.44e−3).

In our evaluation of PRS for quantitative traits such as height and BMI within the KoGES dataset, we applied the R-square metric to gauge the variance explained by the PRS models for these two traits. For the trait of height, our results in Fig. 5 indicate that the DNN+Stacking model achieved the highest R-square value of 0.030, demonstrating its superior predictive power. In contrast, the single DNN models showed a range of R-square values with the model at the p-value threshold of <5e−08 recording the lowest at 0.010, and the model with the threshold of <5e−05 showing a higher value at 0.015. Among the conventional PRS models, PRS-CS with the UKBB European reference panel revealed an R-square value of 0.018, while the PRS-CS with the 1KG East Asian panel displayed a comparable value of 0.017. For BMI, the DNN+Stacking model again stood out with the highest R-square value of 0.035, reaffirming its effectiveness across different phenotypes. The DNN models with more stringent p-value thresholds, specifically the model at p-value <0.0005, showed a substantial R-square value of 0.030, reflecting a better fit than those with higher p-value thresholds. Traditional models like BayesR and P+T showed lower R-square values at 0.010 and 0.015, respectively, which, although indicative of some predictive capability, did not match the performance of the DNN models.

The integration of KoGES data with UKBB has likely contributed to the performance improvements of the models, indicating the benefit of incorporating diverse genetic backgrounds into the prediction models. This integration underscores the versatility and robustness of the DNN+Stacking approach, which not only outperforms the conventional models but also demonstrates the ability to leverage complex patterns within varied datasets for improved predictive accuracy.

## Discussion

Our study focused on enhancing personalised medicine by estimating genetic susceptibility to complex diseases through polygenic prediction. Current methods, though effective, have notable limitations. They fail to consider non-linear interactions of genetic variants due to an underlying additive model assumption^20^ in GWAS, neglecting alternative genetic architectures such as the multiplicative model^21^ and interactions like epistasis^22,23^. The high cost of LD panels, compounded by a need for diversity expansion to include non-European populations, also presents challenges. We demonstrated that DNN models, paired with stacking methods, can effectively manage the volume of genomic data, and more importantly, prevent overfitting. We introduced a novel method of applying stacked DNN for PRS prediction, showing that it can incorporate a broader range of variants compared to conventional models. This results in a more efficient prediction model that can mitigate overfitting. The performance of our DNN model surpassed existing PRS models, indicating a non-linear genetic architecture in the evaluated cancers and quantitative traits. This emphasises the need for non-linear models like DNNs in PRS calculation to improve disease prevention and management.

Similar to our work, prior studies^24,25^ have also utilised deep learning for PRS prediction, using SNPs selected via GWAS. Badre et al.^24^ compared diverse models for breast cancer genetic risk prediction, while Zhou et al.^25^ focused on identifying significant SNPs linked to Alzheimer’s disease. Differently, our study specifically constructs a PRS model using DNN without a fixed p-value threshold, aiming for greater adaptability to various genetic datasets and architectures. Our study emphasises the significance of PRS model regularisation, as demonstrated by the advancements made through stacked DNN. Furthermore, our findings reinforce the potential of using deep learning models to build PRS models, even when utilising a biobank-level database like the UK Biobank, which typically has a comparatively smaller number of cases. This provides valuable insights into the applicability of advanced machine learning methods for genetic risk prediction in diverse population cohorts.

Despite its advantages, DNN also has limitations in predicting PRS. For instance, the SNP inputs for the model, selected via GWAS, may overlook SNPs that don’t display a linear correlation to traits but exert effects nonetheless, restricting the discovery of novel variants. Furthermore, DNN model interpretation remains challenging as prevalent XAI models like LIME^28^ or Grad-CAM^29^ only provide individual feature contributions, not the impact of feature combinations. DNN models also tend to require a large number of samples, posing a challenge for diseases or traits with lower prevalence or a limited number of case samples. These models are unable to effectively use the summary statistics from GWAS meta-analysis studies that boast considerable sample power. This makes the DNN model less feasible for diseases or traits with insufficient case samples, mainly due to the extensive dimension of genomic data relative to the number of available samples. Additionally, the complex LD pattern complicates model training, with the learning of LD information requiring a sizable number of samples.

Given the vast dimensionality of genotype data, any PRS model using raw genotype data could be susceptible to overfitting. GWAS is a beneficial way to reduce the dimension of genomic data for linear models used in conventional PRS models. However, this is predicated on the assumption of independence between each genetic variant, a notion far from the reality. GWAS primarily selects genetic variants linearly correlated with a trait, potentially overlooking variants with non-linear effects. For example, a specific SNP might not demonstrate a significant standalone effect, but certain SNP combinations could. The selection of such SNPs, however, is computationally demanding. An alternative solution might be the extraction or embedding of genetic variant information using machine learning or deep learning approaches. However, genetic variants have unique characteristics that differentiate them from the natural language data that many pre-trained models are developed to handle. For instance, genetic variants typically feature a smaller vocabulary, making it difficult to apply pre-trained models like language or vision models directly to genetic problems without significant modification. This difficulty emphasises the need for a model architecture specifically tailored to understand and learn the particular features of genomic data. Additionally, a genome-specific training method could also be beneficial. Such specialised models and training methods would likely offer improved performance in handling the complexities of genomic data, ultimately contributing to more accurate PRS predictions.

Building on the findings and limitations of this study, we recommend several directions for future research to enhance the PRS prediction model’s effectiveness. This is particularly relevant when dealing with fewer samples, and it includes exploring dimension reduction techniques or pre-training methods. Enhancing the prediction of phenotypic probability with fewer samples could potentially be achieved through dimension reduction techniques or pre-training. The primary challenge here is the complex LD pattern that often confuses models, leading to overfitting. If a pre-trained extractor could effectively condense the input, predicting the polygenic risk score might require fewer samples than a standard DNN model. However, implementing pre-training models in genomics presents its unique hurdles. Unlike natural language or visual data, genetic variants possess distinct characteristics, such as a smaller vocabulary size. This difference complicates the direct application of pre-trained models successful in language or vision domains without necessary modifications for solving genetic problems. Therefore, future research should focus on developing model architectures capable of understanding and learning the unique features of genomic data. Additionally, more investigations should explore genome-specific training methods. These enhancements could lead to more robust and accurate PRS prediction models, potentially improving personalised healthcare outcomes.

In summary, this research highlights the potential of DNN models for improving PRS, though it also acknowledges significant challenges such as interpretation difficulty, sample size requirements, and overfitting due to genomic data’s large dimensionality. However, advances in machine learning and dimensionality reduction could help overcome these issues. Despite limitations, our results demonstrate the promise of these models in personalised medicine and disease management. Further refinement and exploration are required to fully realize this potential.

## Methods

### UK Biobank

PRS models for two highly prevalent and heritable cancers, breast and prostate cancer, were developed utilising genotyped data from the UK Biobank Axiom Array^30^. A cohort of approximately 500,000 participants, aged between 37 and 73 years, was assembled by the UK Biobank from 2006 to 2010. This included comprehensive genotyping and collection of baseline characteristics. Only white British individuals, verified through both self-reported data and genetic evidence, were incorporated into the study. In addition to cancer risk, the study also included quantitative traits such as height and BMI to build the PRS models, utilising the rich dataset provided by the UK Biobank to further understand the genetic risk factors for these cancers.

Restrictions were also put in place to exclude participants with unavailable genetic information, those demonstrating aneuploidy, and those related to either first, second, or third-degree relatives. Written informed consent was obtained from all participants in the UK Biobank, permitting access to their data and samples for research purposes. The study’s ethical approval was granted by the North West Multi-centre Research Ethics Committee, the National Information Governance Board for Health & Social Care, and the Community Health Index Advisory Group, ensuring adherence to all relevant guidelines and regulations throughout the study.

### Korean Genome and Epidemiology Study

In this study, we leveraged the KoGES dataset as a crucial external dataset for validation purposes. The KoGES dataset, renowned for its comprehensive genomic and epidemiological data on a Korean population, provided us with a unique opportunity to validate our PRS models beyond the initial training and GWAS cohort.

Importantly, the KoGES dataset was exclusively used for the validation phase of our PRS models. It was not utilised in any part of the model training or in the GWAS analysis. This approach allowed us to assess the generalisability and predictive performance of our PRS models across different ethnic groups and to ensure that our findings were not merely reflective of the specific characteristics of the initial training cohort. In testing our PRS models, we specifically focused on common genetic variants identified between the UKBB dataset and the KoGES. This approach ensured that the validation of our PRS models was based on a consistent set of genomic markers across both datasets.

The research utilised genomic cohort data from the health examines study (HEXA) within the KoGES^27^ for validation with external dataset. The HEXA study gathered data from 38 healthcare facilities and local screening centres between 2004 and 2013 using consistent protocols. Out of the 65,642 urban individuals surveyed initially and subsequently, epidemiological information came courtesy of the Korea Centres for Disease Control and Prevention.

### Ascertainment of cancer incidence

We utilised hospital episode statistics to identify disease outcomes. Criteria from existing studies^31,32^ guided the identification of cases and controls for the cancers. The extraction of International Classification of Diseases (ICD) codes from hospital admissions (versions ninth and tenth) and self-reported disease data, was done in accordance with advice from previous research^33^.

For UK biobank, the definition of breast cancer cases involved women with a malignant breast neoplasm, as identified via ICD9, ICD10, or self-reporting data. Prostate cancer cases were similarly determined; men presenting with a malignant prostate neoplasm, as ascertained by ICD9, ICD10, or self-reporting data. Those not exhibiting any cancer-associated indications were categorised as controls, with women serving as breast cancer controls and men as prostate cancer controls. The breast cancer sample for this study encompassed 104,425 incidents, including 9286 cases and 95,139 controls. The data set for prostate cancer contained a total of 94,741 individuals, comprised of 7487 cases and 87,254 controls.

For the KoGES dataset specifically, cases of cancer were determined based on self-reporting. This method parallels the approach used in the UKBB, where controls are defined in a manner consistent with UKBB’s standards. The breast cancer cohort consisted of 37,125 participants, including 399 diagnosed cases and 36,725 controls. The prostate cancer dataset included 19,753 individuals, with 39 identified as cases and 19,713 serving as controls.

### Quality control

To minimise genotyping and imputation errors, genotyped data must be subjected to quality control prior to the calculation of the PRS. This quality control process involves several steps for both genotyped and imputed genotype data. Genetic variants are purged if they (1) exhibit missing call rates over 0.05, (2) have an allele frequency under 0.01, or (3) present Hardy-Weinberg equilibrium exact test p-values under 1e−10. Ambiguous SNPs are also discarded, and variants are deduplicated according to their position. Only SNPs that appear in the genotype data and are used in summary statistics are retained. Moreover, for imputed genotype data, any variants with INFO scores under 0.7 are removed.

### GWAS

To identify statistically relevant SNPs across the genome and gauge their effect sizes, we utilised summary statistics from GWAS acquired from a subset of the UK Biobank. The respective GWAS and the number of SNPs are encapsulated in Table 1. To verify and test the PRS models, we divided the dataset into training, validation, and testing subsets. The initial step was to stratify the dataset on the basis of case–control data. Subsequently, we partitioned it into a 9:1 ratio, constituting a training and a test set. In the final stage, the resulting training set was further subdivided into an 8:2 ratio to serve as a training and a validation set. We used the training set to perform GWAS, thereby circumventing any data leakage.

### Baseline models

We focused on three leading PRS methods: P+T, PRSice^34^, and PRS-CS^15^ as the baseline models. Each of these conventional PRS models was formulated using imputed genotype data taken from the UK Biobank Axiom Array. The PRS was evaluated as a linear combination of each variant’s effect size, represented by its beta coefficient, and the count of alleles at that particular position, as per an additive genetic architecture model. Specifically, for an individual i, the PRS is calculated as per Eq. (2), where M denotes the total number of genetic markers used for PRS, $X_{ij}$ signifies the allele count for the jth SNP of the ith individual, and ${\widehat{b}}_{j}$ represents the estimated effect size of the j-th SNP.

2 $$\begin{aligned} {PRS_{i} = \ \sum _{j = 1}^{M}X_{ij}{\widehat{b}}_{j}} \end{aligned}$$

The objective of the P+T method is to identify significant causal variants associated with traits or diseases by pruning SNPs with LD correlation and imposing a p-value cutoff to exclude variants lacking adequate statistical significance. In this study, we executed the P+T model using PLINK^35^, pruning with $r^{2} \ge 0.1$, denoting the correlation between SNPs having a physical distance (or window size) less than 250 kb, and subsequently imposing a threshold using a p-value of 5e−8, as per the standard^36^. The PRS of the P+T model was computed as per Eq. (2), utilising the chosen SNP set and the beta coefficient of GWAS summary statistics without any alterations.

As previously stated, static p-value and pruning parameters may not be ideal, hence, the PRSice model endeavors to discover optimal parameters applied in the P+T process by validating them using metrics iteratively. We recreated the PRSice model using a software package released on GitHub by Choi and O’Reilly^34^, with the default parameter settings. Specifically, during the pruning phase, we set the window size to 250 kb and $R^{2}$ criteria to 0.1. During the thresholding phase, less significant SNPs were discarded using the p-values listed below. We considered threshold $P_{T}$ values from 5e−8 (the standard) to 1 (which includes all SNPs, called the full model) with an interval of 5e−5 in this study. The polygenic score is then evaluated as the sum of the remaining, largely independent SNPs with GWAS association p-values below a threshold $P_{T}$, weighted by their marginal effect size estimates. Finally, the $P_{T}$ value delivering the highest predictive accuracy in a validation dataset is applied, and the model’s performance is assessed using a separate testing set.

BayesR is a Bayesian mixed model regression method employed for PRS construction. The primary motivation behind its development is to simultaneously handle gene discovery, genetic variance estimation from SNP arrays, and phenotype prediction for new samples, ensuring efficiency and increased power. In the BayesR model, the effects in $\beta$ are assumed to come from a mixture of four normal distributions:

$$\begin{aligned} p(\beta _{j}| \pi , \sigma _{g}^{2}) = \pi _{1}N(0, 0 \times \sigma _{g}^{2}) + \pi _{2}N(0, 10^{-4} \times \sigma _{g}^{2}) + \pi _{3}N(0, 10^{-3} \times \sigma _{g}^{2})+ \pi _{4}N(0, 10^{-2} \times \sigma _{g}^{2}) \end{aligned}$$

Here, the weights $\pi$ represent the mixture proportions and are constrained to sum up to one. The variances $\sigma _{g}^{2}$ indicate the effect size. Notably, to incorporate sparseness into the model, both the effect and variance of the first mixture component are set to zero. The model intuitively suggests that a large fraction of the SNPs will have negligible effects, while a smaller fraction may have more substantial effects. The variance $\sigma _{g}^{2}$ represents the additive genetic variance explained by the SNPs. Instead of predetermining a value for $\sigma _{g}^{2}$, BayesR estimates a hyper-parameter for this genetic variance directly from the dataset. We set the mixture proportion $\pi$ as (0.95, 0.03, 0.01, 0.01), respectively. The BayesR model was executed using the GCTB software, accessible to the public.

The PRS-CS model determines PRS by utilising the beta coefficients of GWAS and also by adjusting the beta coefficients through a technique known as continuous shrinkage. This method alters the distribution of the beta coefficients using a predefined prior distribution and the LD reference. The PRS-CS model takes into account the following phenotype model:

3 $$\begin{aligned} { y = Z\beta + \epsilon ,\ \ \epsilon \sim N( 0,\sigma ^{2}I ),\ \ p( \sigma ^{2} ) \propto \sigma ^{- 2},} \end{aligned}$$

The prior distribution is grounded on global-local scale mixtures of normals as exhibited in Eq. (4), where $\sigma ^{2}$ represents the variance of $\beta _{j}$, N is the sample count, $\phi$ is a global scaling parameter influencing the effect size of all variants, $\psi _{j}$ is a local and marker-specific parameter, and g is an absolutely continuous mixing density function.

4 $$\begin{aligned} {\beta _{j}\sim N( 0,\ \frac{\sigma ^{2}}{N}\phi \psi _{j} ),\ \psi _{j}\sim g,} \end{aligned}$$

The posterior mean of $\beta$ is

5 $$\begin{aligned} {E\left[\beta \big | \widehat{\beta } \right]= \left( D + T^{- 1} \right) ^{- 1}\widehat{\beta },} \end{aligned}$$

where $T = diag\{ \phi \psi _{1},\phi \psi _{2},\ldots ,\phi \psi _{M} \}$ is a diagonal matrix and $D = Z^{T}Z/N$ is the LD matrix, with Z being an $N \times M$ standardised genotypes matrix. The local shrinkage parameter $\psi _{j}$ was assigned an independent gamma–gamma prior as

6 $$\begin{aligned} {\psi _{j}\sim G( a,\delta _{j} ),\ \ \delta _{j}\sim G(b,1)} \end{aligned}$$

The PRS-CS was executed using the software package released on GitHub by Ge et al.^15^.

### Deep neural network

In our study, we introduce a DNN model aimed at predicting genetic risk. This model uses summary statistics for the selection of a significant set of SNPs. A range of DNN architectures were trained and assessed using a grid search approach. This technique helps to determine the most optimal hyperparameters and architecture for the DNN model, which leads to the highest validation score. For every hidden layer neuron, we applied the Leaky Rectified Linear Unit activation function^37^ (Leaky ReLU), as described below:

7 $$\begin{aligned} { LeakyReLU(x) = {\left\{ \begin{array}{ll} x &{} \text {if }\ x \ge 0 \\ \alpha x &{} \text {otherwise.} \end{array}\right. } } \end{aligned}$$

For the output layer, we applied the logistic function, as detailed here:

8 $$\begin{aligned} {Logistic(x) = \frac{1}{1+e^{-x}} } \end{aligned}$$

The loss function was evaluated using the binary cross-entropy (BCE) function for qualitative traits, as defined here:

9 $$\begin{aligned} {BCE = -\frac{1}{N}\sum _{i = 1}^{N}{y_{i}\log (h(x_{i};\theta ))+ (1-y_{i})\log (1-h(x_{i};\theta ))}} \end{aligned}$$

Here, $y \in \{ 0,1 \}$ represents the prediction target, with 1 standing for cases and 0 for controls. $h(x_{i};\theta ) \in [0,1]$ is the predictive probability from the model for the target, given the input $x_{i}$ and its parameters $\theta$. The predicted probability is interpreted as the PRS projected by the DNN.

For quantitative traits, mean squared error (MSE) loss function was used to evaluate as defined here:

$$\begin{aligned} MSE = \frac{1}{N} \sum _{i=1}^{N}(y_{i} - \hat{y}_{i})^{2} \end{aligned}$$

where $\hat{y}$ is predicted value from the model and $y$ is the target value.

We used the Adam optimiser^38^ as the DNN model optimiser, an adaptive learning rate optimisation algorithm. The starting learning rate was established at 1e−3. To diminish the likelihood of overfitting, we implemented Dropout^39^ with a rate of 0.5. We also employed Batch Normalisation (BN)^40^ to improve the training of DNN models by minimising internal covariate shift. When applicable, Dropout and BN were implemented to all hidden layers of the DNN models.

The grid search method tested a series of DNN models that differed in the depth and width of hidden layers. The depth varied from 1 to 4, and the width was decided by dividing the number of outputs from the preceding layer by a number from 1 to 4, which may be different from the number of selected SNPs for input. The tested hyperparameter combinations in grid search experiments encompassed models both with and without regularisation: BN and Dropout. To quicken the grid search process, early stopping was used, which ceases training when performance improvement stagnates, thus saving computational time.

The SNP set, employed as input for the DNN model, was selected based on p-values within a designated range. As the scale of the p-value for summary statistics is dependent on the dataset used in GWAS, a set of p-values was manually created and chosen for each study and disease.

In the development of the DNN model for the KoGES dataset, clumped SNPs were employed as a crucial step in the preprocessing. The rationale behind this choice lies in the inherent nature of the KoGES dataset, which exclusively provided imputed data. Imputed datasets, as opposed to array datasets, often present a heightened level of LD correlations. This extensive LD correlation can introduce redundancies and noise in the data, potentially impacting the model’s performance.

The training and validation of the DNN models mirrored the conventional models. The performance of the DNN models was validated using the average ROC AUC and Nagelkerke R-square for qualitative traits of test sets. Similarly, R-square is used for quantitative traits. The most effective model for each p-value threshold was applied as the base model for the stacking method, which is discussed in the next section. Unlike the PRSice model, the DNN model does not employ its beta coefficient, meaning internal GWAS summary statistics can be used to select the SNP set without the threat of overfitting. Python3 and PyTorch were used to implement the grid search and DNN models.

### SNPRS

The process of selecting specific hyperparameters is inherently empirical, and the optimal set can differ from one dataset to another, potentially introducing bias. To address this, we make use of stacking, an ensemble machine learning technique that combines multiple models trained with a variety of hyperparameters. In this study, we’ve applied this technique to mitigate potential bias arising from hard thresholding when building a PRS model based on deep-learning approaches. The overall structure of our SNPRS model is illustrated in figure Fig. 1. The detailed steps of constructing the SNPRS are provided in Algorithm 1.

The procedure for constructing the SNPRS involves building multiple base models using DNNs and then aggregating these base models, denoted $\hat{h}_{t}$, using a meta learner, denoted $\hat{h'}$. The initial step requires creating a collection of significant sets of variants, $Z_{1:T}$, based on a range of p-value thresholds $\textbf{p}$, to serve as inputs for the base models. Once multiple groups of variants have been selected, we find the optimal hyperparameters for the DNN model using both the training and validation datasets.

Subsequently, we create the input for the meta learner, $\hat{\textbf{y}}$, by concatenating the outputs of $\hat{h}_{1:T}(Z_{1:T})$ using the validation dataset. These outputs are used to train the meta learner, a straightforward linear model designed to predict the final results. Ultimately, we evaluate the performance of the SNPRS model, $\hat{H}(Z_{1:T})$, using the test dataset. This procedure efficiently addresses potential biases in the selection of hyperparameters, thereby providing a more robust and reliable prediction of genetic risk.

**Algorithm 1:**
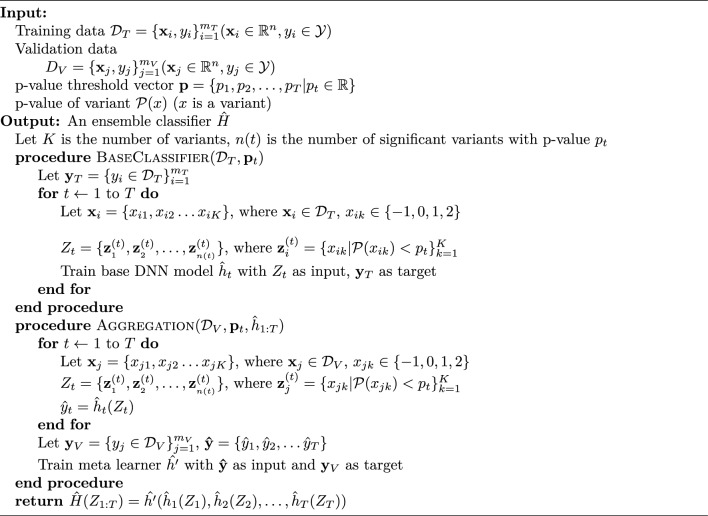
Stacked neural network.

### Supplementary Information

Supplementary Information.

## Data Availability

The PRS-CS algorithm, accessible at https://github.com/getian107/PRScs, served as a baseline model and was implemented using Python3. Similarly, another baseline model was the PRSice algorithm, built using R and accessible at https://github.com/choishingwan/PRSice. The majority of the genotype data preprocessing was conducted using PLINK2, which can be found at https://www.cog-genomics.org/plink/2.0/. For the implementation of the BayesR model within our study, we utilised the GCTB software, which is publicly available at https://cnsgenomics.com/software/gctb/#Overview.
