# Supplementary material for: Stacked neural network for predicting polygenic risk score
Source: Sci Rep. 2024 May 21;14:11632. doi: 10.1038/s41598-024-62513-1 (PMC11109142; doi:10.1038/s41598-024-62513-1)
Supplement: Supplementary file 1 — Supplementary Information. [file 41598_2024_62513_MOESM1_ESM.pdf]

Stacked Neural network for predicting Polygenic Risk Score

Sun bin Kim[1] Joon Ho Kang[1] MyeongJae Cheon[1] Dong Jun Kim[1]
Byung-Chul Lee[1,*]

Feb 29th, 2024

[1] Genoplan Korea Inc., Seoul, Republic of Korea

[*] Correspondence: Byung-Chul Lee <io@genoplan.com>

Supplementary Document

GWAS Results

It's important to note the disparity between the figures for the imputed and array datasets. The Q-Q plot for the imputed dataset revealed a denser spread of points, indicating the inclusion of additional genetic variants informed by LD. Overall, the results from our GWAS analysis on the training set, utilizing both UKBB array data for DNN and SNPRS, and imputed UKBB dataset for conventional models, affirm the successful identification of genetic associations with the traits of interest.

PRS Results

Supplemenetary Table 1: Simulation Studies Results Conducted with GCTA

polygenicity	heritability	prevalence	num_case	num_ctrl	prsice	prs_cs_1kg	prs_cs_ukbb	DNN
0.001	0.3	0.01	100	100	0.52375	0.547	0.536	0.572483025
0.01	0.3	0.01	100	100	0.49375	0.5605	0.56	0.597815286
0.1	0.3	0.01	100	100	0.49525	0.5085	0.5085	0.556999228
0.2	0.3	0.01	100	100	0.50125	0.5165	0.5225	0.550859997
0.001	0.3	0.05	100	100	0.53025	0.5495	0.5375	0.590538194
0.01	0.3	0.05	100	100	0.51925	0.5025	0.5145	0.570363855
0.1	0.3	0.05	100	100	0.52	0.517	0.516	0.561764275
0.2	0.3	0.05	100	100	0.53425	0.529	0.5515	0.559383145
0.001	0.3	0.01	500	500	0.6396	0.67129	0.67348	0.656992982
0.01	0.3	0.01	500	500	0.6213	0.64408	0.64192	0.656794796
0.1	0.3	0.01	500	500	0.63792	0.65446	0.6578	0.613008535
0.2	0.3	0.01	500	500	0.64294	0.6771	0.68176	0.606216965
0.001	0.3	0.05	500	500	0.57191	0.62652	0.63016	0.587348284
0.01	0.3	0.05	500	500	0.55459	0.59196	0.59646	0.60012749
0.1	0.3	0.05	500	500	0.57071	0.58152	0.58572	0.589588652
0.2	0.3	0.05	500	500	0.54038	0.56034	0.5627	0.575350249
0.001	0.3	0.01	2500	2500	0.7185544	0.7169208	0.7386608	0.741527737
0.01	0.3	0.01	2500	2500	0.655888	0.7033832	0.70332	0.632701375
0.1	0.3	0.01	2500	2500	0.6559016	0.6967252	0.696598	0.6076008
0.2	0.3	0.01	2500	2500	0.6729144	0.7253136	0.7252476	0.643235256
0.001	0.3	0.05	2500	2500	0.6844596	0.6986768	0.6982308	0.70322576

polygenicity	heritability	prevalence	num_case	num_ctrl	prsice	prs_cs_1kg	prs_cs_ukbb	DNN
0.01	0.3	0.05	2500	2500	0.6159616	0.6747176	0.6749636	0.613887242
0.1	0.3	0.05	2500	2500	0.6190968	0.6415788	0.643434	0.558524428
0.2	0.3	0.05	2500	2500	0.6049344	0.6424892	0.6423544	0.552318041
0.001	0.3	0.01	5000	5000	0.759572904	0.761924701	0.764613323	0.714714849
0.01	0.3	0.01	5000	5000	0.693184731	0.721285479	0.727386527	0.650682067
0.1	0.3	0.01	5000	5000	0.688745958	0.726175449	0.724313473	0.595560813
0.2	0.3	0.01	5000	5000	0.683802395	0.71619506	0.716865719	0.610344158
0.001	0.3	0.05	5000	5000	0.7325314	0.7361453	0.7398485	0.729835594
0.01	0.3	0.05	5000	5000	0.6505662	0.6816973	0.6816821	0.580388277
0.1	0.3	0.05	5000	5000	0.6380516	0.6748596	0.6718496	0.577499934
0.2	0.3	0.05	5000	5000	0.6360716	0.6688666	0.6698992	0.591327993

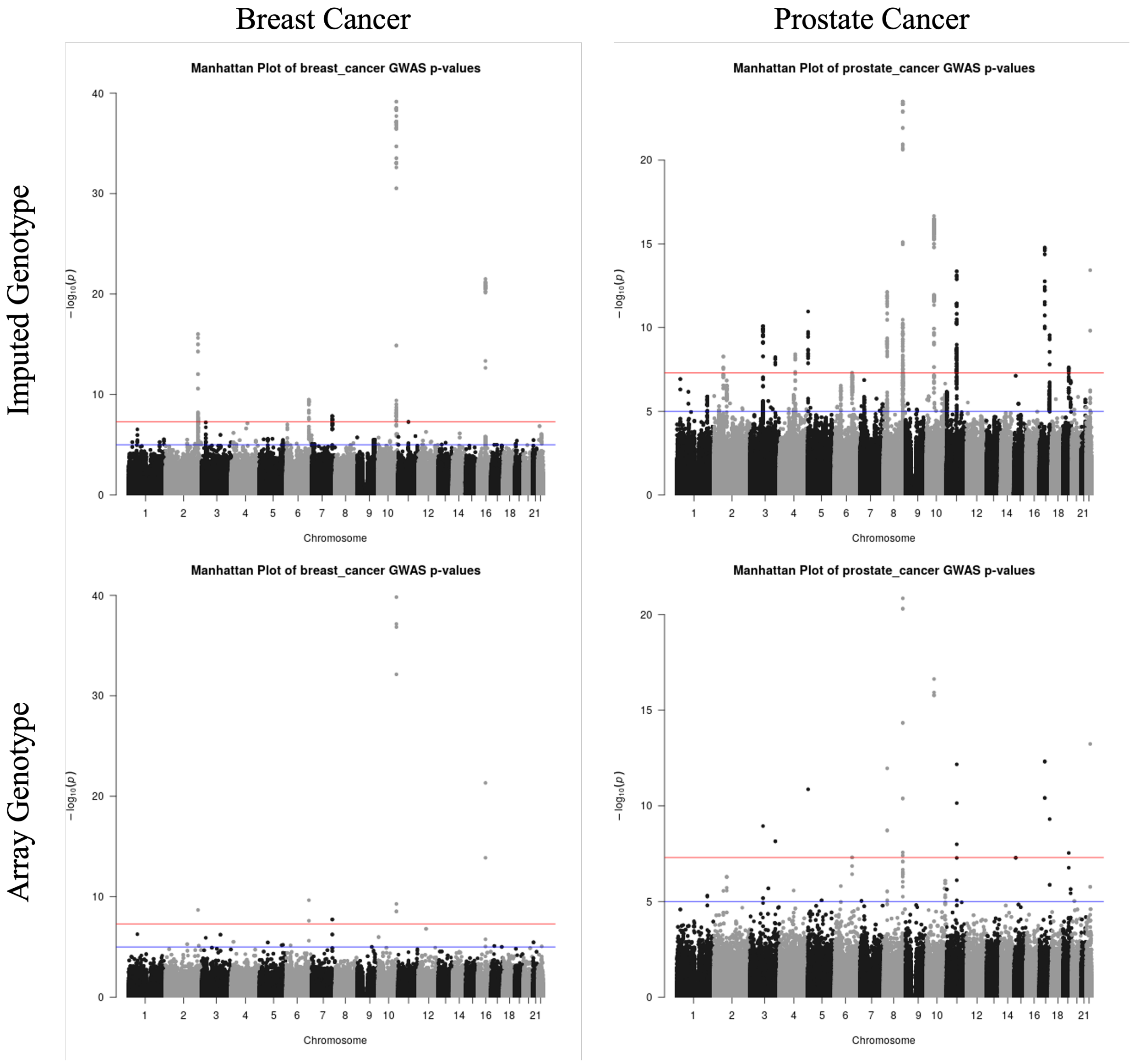

Supplementary Figure 1: Manhattan Plot for the conducted GWAS

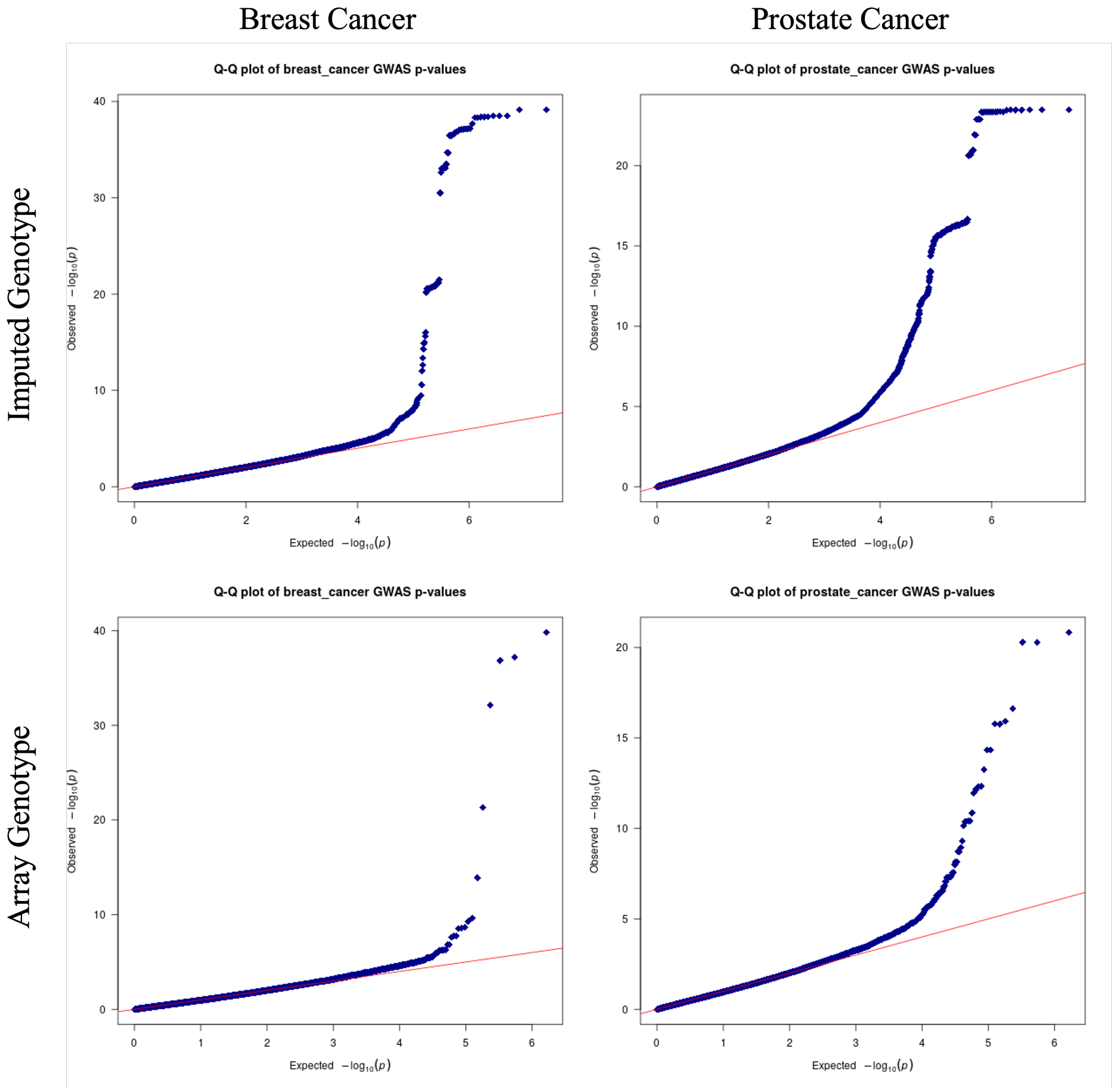

Supplementary Figure 2: Q-Q plots for the conducted GWAS

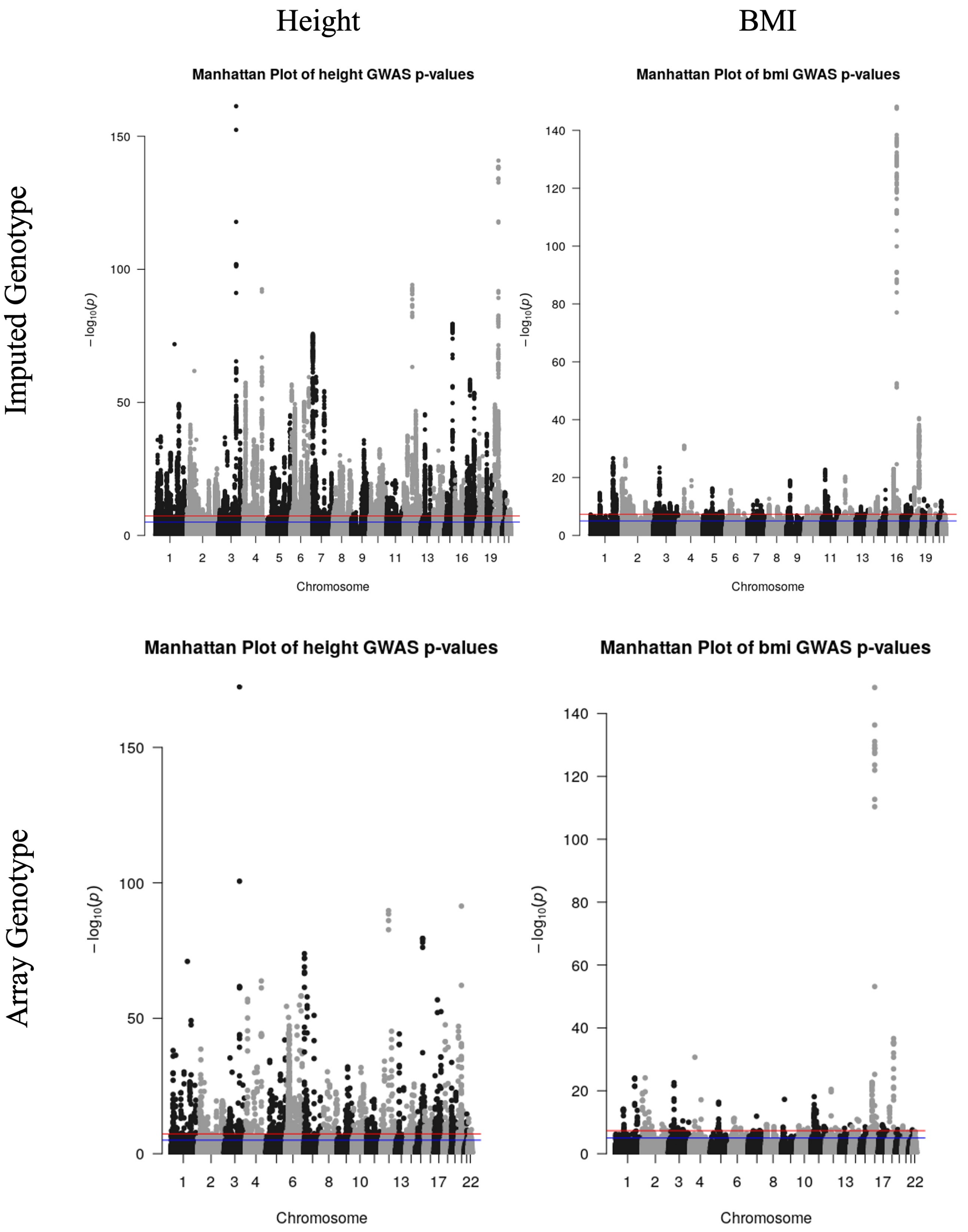

Supplementary Figure 3: Manhattan Plot Obtained from Quantitative Traits in UKBB

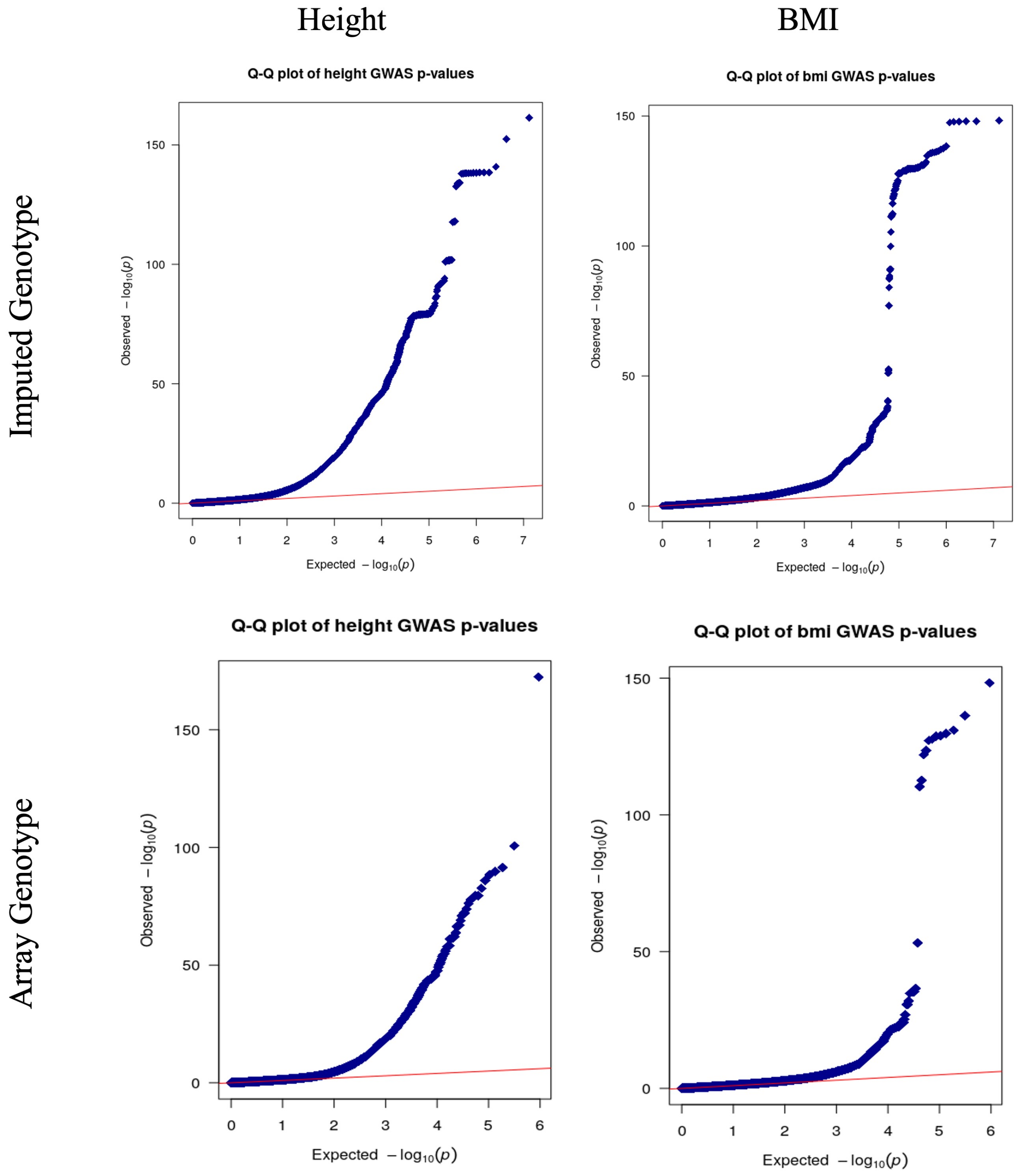

Supplementary Figure 4: Q-Q Plot Obtained from Quantitative Traits in UKBB

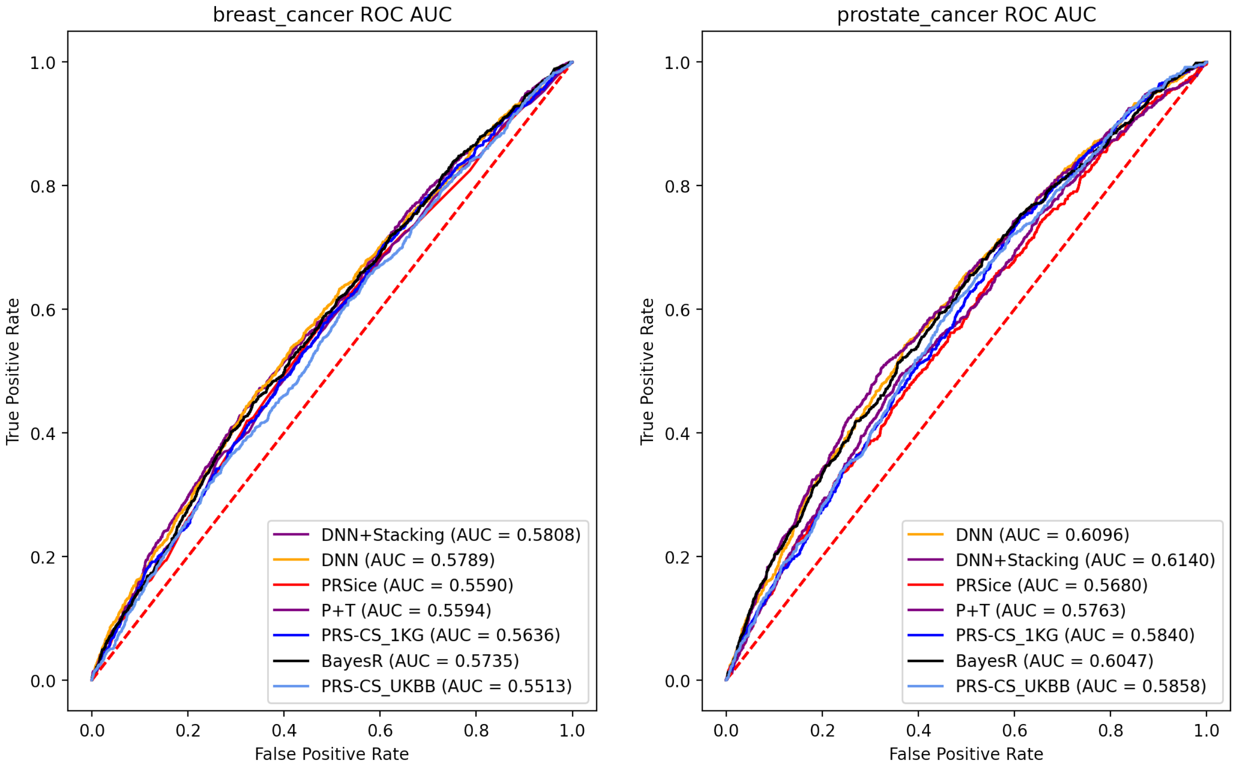

Supplementary Figure 5: Gender-specific Cancers AUC Plot Comparison for UKBB dataset with Other Models

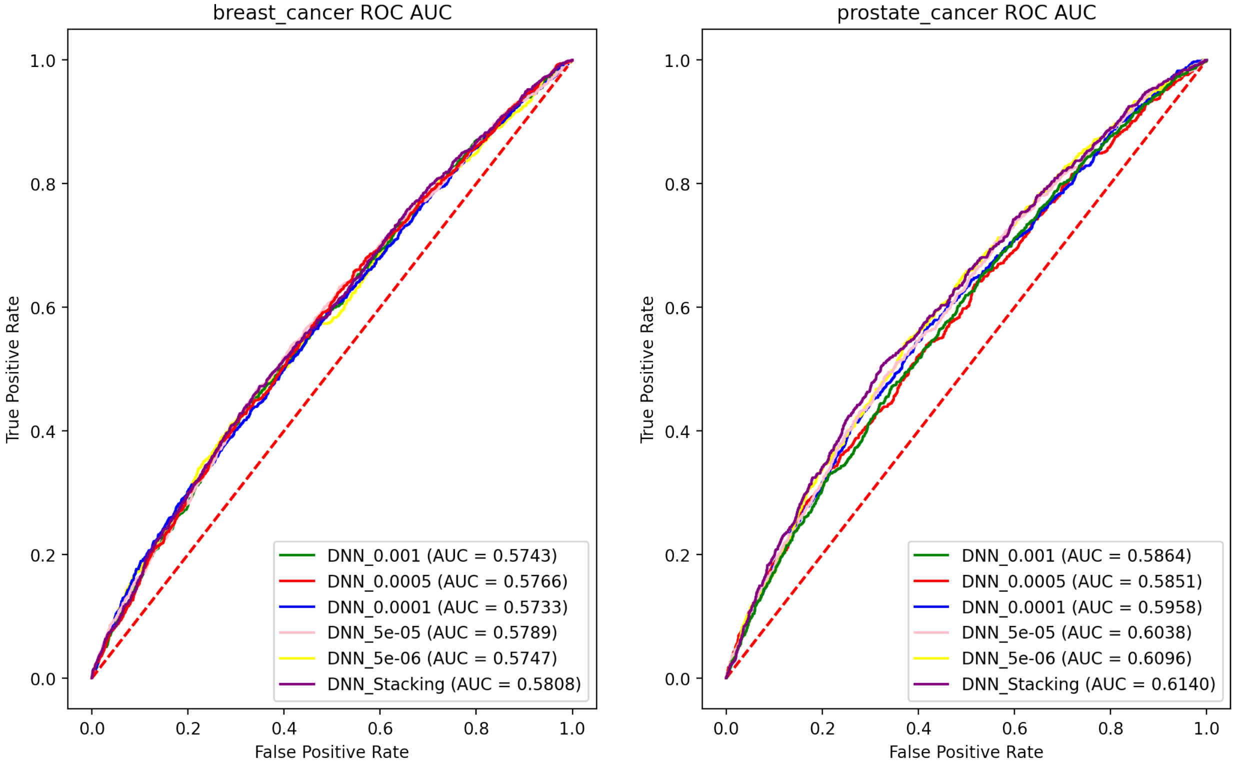

Supplementary Figure 6: Gender-specific Cancers AUC Plot Comparison for UKBB dataset with Single Models and Stacked one

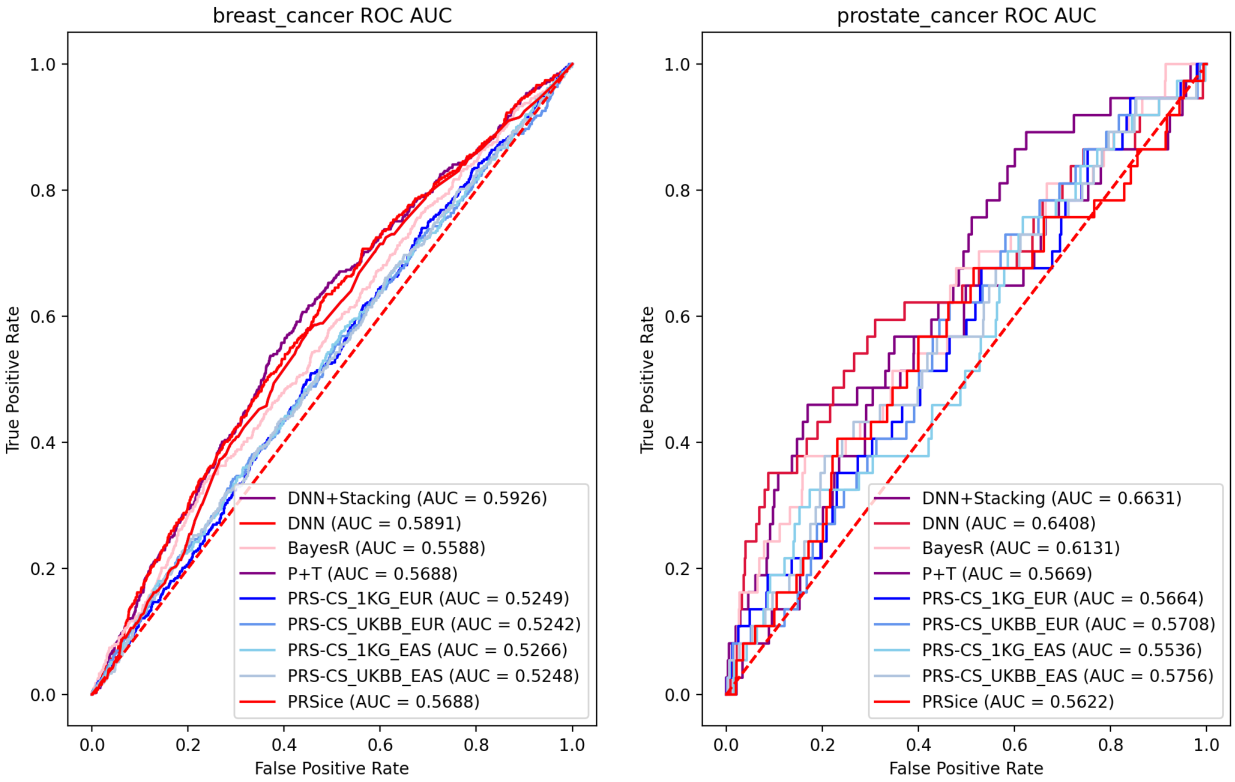

Supplementary Figure 7: Gender-specific Cancers AUC Plot Comparison for KoGES dataset with Other Models

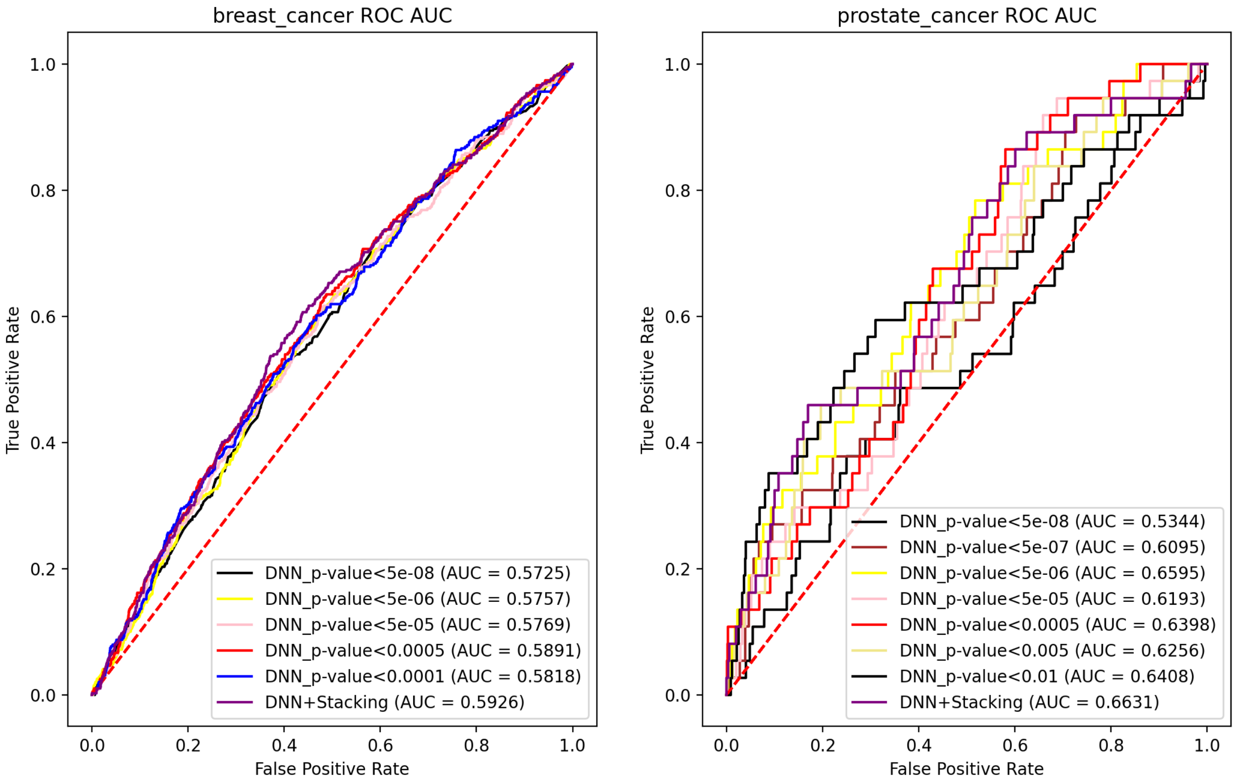

Supplementary Figure 8: Gender-specific Cancers AUC Plot Comparison in KoGES dataset with Single Models and Stacked one
